# Assessing the diagnostic impact of blood transcriptome profiling in a pediatric cohort previously assessed by genome sequencing

**DOI:** 10.1038/s41525-025-00505-4

**Published:** 2025-07-01

**Authors:** Huayun Hou, Kyoko E. Yuki, Gregory Costain, Anna Szuto, Sierra Barnes, Arun K. Ramani, Alper Celik, Michael Braga, Meagan Gloven-Brown, Dimitri J. Stavropoulos, Sarah Bowdin, Ronald D. Cohn, Roberto Mendoza-Londono, Stephen W. Scherer, Michael Brudno, Christian R. Marshall, M. Stephen Meyn, Adam Shlien, James J. Dowling, Michael D. Wilson, Lianna Kyriakopoulou

**Affiliations:** 1 Program in Genetics and Genome Biology, SickKids Research Institute, Toronto, ON Canada; 2 Division of Genome Diagnostics, The Hospital for Sick Children, Toronto, ON Canada; 3 Division of Clinical and Metabolic Genetics, The Hospital for Sick Children, Toronto, ON Canada; 4 Department of Paediatrics, Temerty Faculty of Medicine, University of Toronto, Toronto, ON Canada; 5 Department of Molecular Genetics, University of Toronto, Toronto, ON Canada; 6 Centre for Computational Medicine, The Hospital for Sick Children, Toronto, ON Canada; 7 Department of Laboratory Medicine and Pathobiology, University of Toronto, Toronto, ON Canada; 8 Department of Clinical Genetics, Cambridge University Hospitals NHS Foundation Trust, Cambridge, UK; 9 Institute of Medical Science, University of Toronto, Toronto, ON Canada; 10 The Centre for Applied Genomics, The Hospital for Sick Children, Toronto, ON Canada; 11 University Health Network, Toronto, ON Canada; 12 Department of Computer Science, University of Toronto, Toronto, ON Canada; 13 Vector Institute for Artificial Intelligence, Toronto, ON Canada; 14 Center for Human Genomics and Precision Medicine, University of Wisconsin, Madison, WI USA; 15 Department of Pediatrics, University of Wisconsin, Madison, WI USA; 16 Division of Neurology, The Hospital for Sick Children, Toronto, ON Canada

**Keywords:** Genetics research, Gene expression analysis, Clinical genetics, Genetic testing

## Abstract

Despite advances in genome sequencing, many individuals with rare genetic disorders remain undiagnosed. Transcriptional profiling via RNA-seq can reveal functional impacts of DNA variants and improve diagnosis. We assessed blood-derived RNA-seq in the largely undiagnosed SickKids Genome Clinic cohort (*n* = 134), which has been subjected to multiple analyses benchmarking the utility of genome sequencing. Our RNA-centric analysis identifies gene expression outliers, aberrant splicing, and allele-specific expression. In one-third of diagnosed individuals (20/61), RNA-seq reinforced DNA-based findings. In 2/61 cases, RNA-seq revised diagnoses (*EPG5* to *LZTR1* in an individual with a Noonan syndrome-like disorder) and discovered an additional relevant gene (*CEP120* in addition to *SON* in an individual with ZTTK syndrome). Additionally, ~7% (5/73) of undiagnosed cases had at least one plausible candidate gene identified. This study highlights both the benefits and limitations of whole-blood RNA profiling in refining genetic diagnoses and uncovering novel disease mechanisms.

## Introduction

Exome and genome sequencing (GS) technologies have transformed our ability to identify causal genetic variants and make diagnoses for rare diseases (e.g^1^.; reviewed by refs. ^2,3^). Despite this progress, more than half of individuals with a genetic disorder remain without a diagnosis. This can be partly attributed to our limited understanding of the downstream consequences of genetic variation and the lack of functional evidence for its interpretation. RNA sequencing (RNA-seq) has the potential to identify clinically relevant aberrations and provide a high throughput functional platform for those variants related to RNA expression levels and/or RNA processing.

Several studies have used transcriptome profiling to assess its potential diagnostic utility in rare disorders^4–15^. For example, RNA-seq on muscle biopsies identified molecular diagnoses in up to 35% of individuals with neuromuscular disorders^8,9,12^. RNA-seq using fibroblasts yielded a diagnostic rate of 10–16% in individuals with mitochondrial diseases^13,14^. Fresard et al. (2019) demonstrated that RNA-seq in whole blood increased the absolute diagnostic rate by 7.5% in a phenotypically heterogeneous cohort with suspected rare diseases^11^. While these studies support the ability of RNA-seq to improve diagnostic rates, there are few studies benchmarking the RNA-seq yield when performed as an adjunct to genome sequencing^10^.

In this study, we assessed the utility of RNA-seq for decreasing uncertainty and increasing the yield of GS-based diagnoses. To do this we performed RNA-seq on RNA isolated from peripheral blood samples donated by children and youth with suspected genetic disorders who enrolled in the Genome Clinic at the Hospital for Sick Children (Toronto, ON, Canada). This longitudinal study was designed to test the diagnostic and predictive use of GS^16,17^. The participants presented with a spectrum of complex clinical phenotypes such as epilepsy, global developmental delay and multiple congenital anomalies and had previously been tested by standard of care genetic testing. After genome sequencing and subsequent reanalyses, the diagnostic rate was 41%^16–18^. Given the clinical work up and intense focus on genome analysis in this cohort, we reasoned that performing RNA-seq on all individuals who provided blood samples would be an important and unique opportunity to examine the concordance between genome-based diagnoses and RNA-seq as well as ask whether new potential candidate disease genes related to the individual’s phenotype could be revealed by RNA-seq. In this study we focused on two clinically meaningful scenarios, where we asked if transcriptomic data could: (1) provide functional evidence for several classes of suspected/known diagnostic genetic variants identified by GS; and (2) identify plausible candidate genes for individuals for whom GS was initially non-diagnostic.

## Results

The cohort for this study consisted of all individuals from the SickKids Genome Clinic for whom a primary blood sample was available (84/100 from Stavropoulos et al. 2016^16^ and 50/103 from Lionel et al.^17^). This provided us with a cohort of 134 participants (75 males and 59 females) between 0 and 18 years of age at the time of sample collection (median 6 years; Supplementary Data 1). These individuals displayed a wide array of symptoms described by 453 unique HPO terms across the cohort. The most observed phenotypes were epilepsy (29%), global developmental delay (25%), multiple congenital abnormalities (19%), connective tissue (5%), eye (6%), behavior/cognition - other (3%), neurological - other (2%), immune (2%), cardiovascular (1%) and metabolic including mitochondrial (9%); Table 1 and Supplementary Data 2).

**Table 1:** Transcriptional impact of disease genes detected in blood

Case	Dx	Diagnosis Description	Variant	Origin	Transcriptional impact	Variant classification	Reference
11	Y	Thiamine metabolism dysfunction syndrome 4 (613710)	*SLC25A19* c.495 G > A p.(Met165Ile) (het) [NM_001126122.1]; chr17:(73,267,001-73,271,500)x1	N/A	NM_001126122.1:r.?_774:: NM_020679.4:r.-50_? decreased expression zScore: -4.09, FC: 0.68, *p* value: 1.77e-4	Likely pathogenic; Pathogenic^	Lionel et al^17^.
19#	Y	Infantile epileptic encephalopathy; Severe global developmental delay	*PPP3CA* (NM_000944.4): c.1299dupC (p.Ser434Glnfs*17)	DN	Decreased expression zScore: -3.95, FC: 0.77, *p* value: 1.15e-04	Pathogenic	
38#	Y	Bardet-Biedl syndrome (BBS)	*BBS1* c.1169 T > G (p.Met390Arg) (het) [NM_024649.5]; *BBS1*:NM_02649.4:c.1214–1215ins (1700_1800);1198_1214	M/P	Decreased expression zScore: -3.88, FC: 0.65, *p* value: 4.60e-05	Pathogenic; Pathogenic	Tavares et al.^20^
43	Y	Mental retardation, autosomal dominant 19 (615075)	*CTNNB1* c.1041_1044delATCT (p.Val349Alafs*9) (het) [NM_001904.3]	DN	Decreased expression zScore: -6.34, FC:0.67, *p* value: 9.24e-10	Pathogenic	Lionel et al.^17^
46	Y	3-Methylglutaconic Aciduria, Type V (610198)	*DNAJC19* c.280+1_280+5delGTAAG (p.?) (hom) [NM_145261.3]	N/A	r.130_280del and r.210_280del; decreased expression zScore: -5.98, FC:0.61, *p* value = 3.36e-9	Likely pathogenic^	Lionel et al.^17^
59	Y	Infantile Sialic Acid Storage Disease (269920)	*SLC17A5* c.291 G > A (p.Thr97 = ) (het); c.819+1 G > A (p.?) (het); in trans [NM_012434.4]	N/A	r.95_291del and r.701_819del; decreased expression: zScore: -3.84, FC: 0.63, *p* value 1.56e-4	Pathogenic^; Pathogenic^	Lionel et al.^17^
86	Y	Congenital disorder of glycosylation, type IIm (300896)	*SLC35A2* c.991 G > A (p.Val331Ile) (het) [NM_005660.1]	DN	allelic imbalance towards reference allele Ref 45:, Alt:14, padj: 0.5	Pathogenic	Lionel et al.^17^
1008	Y	Coffin-Siris syndrome	*SMARCB1* c.364del (p.Glu122Asnfs*21) (het) [NM_003073.3]	N/A	r.363_500del	Likely pathogenic^	Stavropolous et al.^16^
1009	Y	Alazami Syndrome	*LARP7* c.756_757del (p.Arg253Ilefs*6) (hom) [NM_016648.2]	M/P	Decreased expression zScore: -7.77, FC: 0.37, *p* value: 2.53e-13	Pathogenic	Stavropolous et al.^16^
1016	Y	Neurodegeneration with brain iron accumulation-1	*PANK2* c.824_825del (p.Cys276Trpfs*15) (hom) [NM_153638.2]	M/P	r.839_981del	Pathogenic^	Stavropolous et al.^16^
1022	Y	10p11.23-p11.2 deletion	arr 10p11.23p11.22(30,822,400-32,872,150)x1	DN	Decreased expression	Pathogenic	Stavropolous et al.^16^
1026	Y	22q12.3 deletion	arr 22q12.2 (35,931,002-37,272,620)x1	N/A	Decreased expression	Pathogenic	Stavropolous et al.^16^
1027	Y	16p13.11 deletion	arr 16p13.11(15,507,164-16,400,833)x1	DN	Decreased expression	Pathogenic	Stavropolous et al.^16^
1034	Y	22q11.2 Deletion syndrome	arr 22q11.21(18,713,432-21,440,515)x1	DN	Decreased expression	Pathogenic	Stavropolous et al.^16^
1049	Y	Sotos syndrome	*NSD1* c.3922-1 G > C (p.?) (het) [NM_022455.4]	N/A	r.3922_3926del and r.3922_4192del	Pathogenic^	Stavropolous et al.^16^
1066	Y	Cerebral cavernous malformation and 8q22.1 deletion.	*CCM2* c.1054delG (p.Gly352Valfs*2) (het) and arr 8q22.1(97,145,564-98,301,541)x1	SNV: P; CNV: DN	Decreased expression of 8q22.1 genes	Pathogenic; Pathogenic	Stavropolous et al.^16^
1089	Y	Diarrhea 10, protein-losing enteropathy type	*PLVAP* c.1072 C > T (p.Arg358*) (hom) [NM_031310.1]	M/P	Decreased expression zScore: -6.56, FC: 0.03, *p* value: 2.27e-6	Pathogenic	Stavropolous et al.^16^
1090	Y	Turner syndrome	arr Xp22.33q21.32(60,701-91,873,757)x3, Xq21.32q28(91,877,172-155,174,078)x1	DN	Genes in region have altered expression	Pathogenic	Stavropolous et al.^16^
1103	Y	Pontocerebellar hypoplasia, type 2E	*VPS53* c.1429 C > T (p.Arg477*); c.1716 T > G (p.Ser572Arg) [NM_018289]	M/P	Allelic imbalance towards nonsense variant containing reference allele Ref 39:, Alt: 12, padj: 0.5 Ref 12:, Alt: 39, padj: 0.3	Likely pathogenic;	Stavropolous et al.^16^
1058*	Y	ZTTK syndrome	*SON* c.3476delC (p.Pro1159Argfs*9) (het) [NM_138927.2]	DN	Decreased expression, zScore: -3.50, FC: 0.80, *p* value: 6.00e-4 also detect altered splicing in CEP120 NM_153223.4 r.2481_2482ins2482-2888_2482-3106	Pathogenic	Costain et al.^18^

The case ID, presence of a diagnosis (Dx). Cases included in cohort from Lionel et al.^17^. are designated by #; Cases included in the cohort from Stavropolous et al.^16^. are designated with *; *het* heterozygous, *hom* homozygous, Origin of mutation abbreviations: *M* maternal, *P* paternal, *DN* de novo, *N/A* not available; ^ indicates use of pathogenic very strong (PVS1) criteria as per ACMG guidelines for splicing^71^ following RNA-seq analysis.

Based on prior published analyses, we split this cohort into two groups: Group 1 consisted of 61 individuals (35 male and 26 female) who received a diagnostic DNA variant(s) in the initial GS-based studies^16–18^. These variants were classified as pathogenic/likely pathogenic as per ACMG guidelines^19^ and were considered causative of the patient’s clinical phenotype. Group 2 consisted of the remaining 73 individuals (40 male and 33 female), who had yet to receive a molecular genetic diagnosis. The logic behind partitioning this well studied cohort into two groups was that it would allow us to: (a) assess the relevance of RNA-seq alone in identifying/corroborating existing diagnoses (Group 1); and (b) suggest genes and possible pathogenic variants related to the primary indication for referral in the unsolved cases (Group 2).

### Developing and applying a scalable and reproducible RNA-seq library preparation method to 134 whole blood samples

**Fig. 1:**
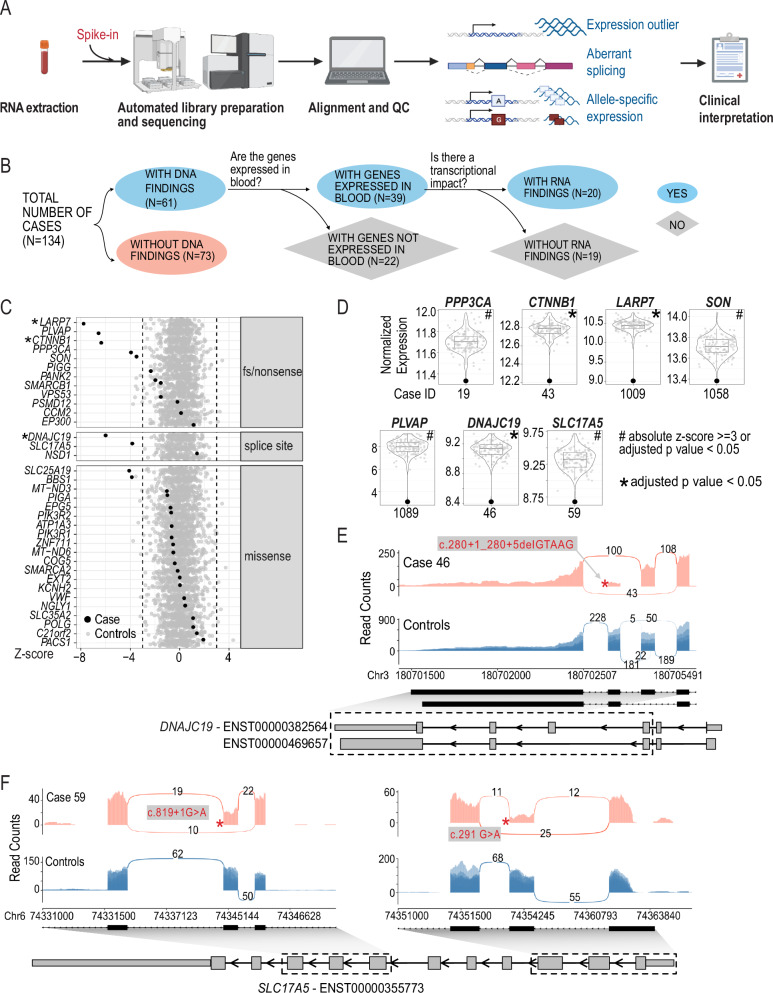
Effects of pathogenic or likely pathogenic SNVs and indels on gene expression. **A** RNA-seq-based clinical diagnosis workflow. **B** Flowchart showing the breakdown of samples. Created in BioRender. Wilson, M. (2025) https://BioRender.com/y35t706. **C** Detection of genes with diagnostic variants as expression outliers. Each row represents one gene. Each dot represents the z-scores of the corresponding gene in all samples. Black: cases in which the corresponding gene had a diagnostic variant; Grey: rest of the cohort as control samples. Asterisks indicate that the candidate gene is also detected as significant (adjusted *p* value < 0.05). Dashed lines indicated z-score of –3 or 3. Genes are separated into different panels based on the type of mutations they harbor. **D** Violin and boxplot showing normalized gene expression of selected candidate genes across the cohort. Y axis: log2 normalized read counts of the target gene in all samples in the cohort. Each dot represents one sample. Highlighted dot represents the case of interest. Case IDs are shown below each plot. The gene is considered an outlier in the analysis is designated by ‘#’ when absolute z-score >=3 or adjusted *p* value < 0.05) or by ‘*’ when the gene is detected as significant outlier with an adjusted *p* value < 0.05. **E** Impact of pathogenic variants in *DNAJC19* on splicing and gene expression. **F**. Impact of pathogenic variants in *SLC17A5* on splicing and gene expression. Sashimi plots show reads and junctions in the affected region for case (red track) and control samples (blue track, *n* = 10, randomly selected from the cohort) with the DNA variant labeled with an asterisk. Structures of relevant transcripts are shown with UTRs shown as thinner boxes. Only UTRs of transcripts annotated as “protein-coding” are shown. Exons shown in the sashimi plots are highlighted with a dashed box.

Compared to genome sequencing, considerable technical variability can occur when performing RNA-seq. Our long-term objective is to establish a clinically validated RNA-seq procedure that can run in our clinical lab so that RNA-seq samples prepared over extended periods of time will show minimal batch effects. We established a semi-automated RNA-seq library preparation protocol with a workflow suitable for clinical laboratory testing. The sample preparation protocol included synthetic spike-in controls that allow us to assess the quality of the library preparation (ERCC + SIRV controls; see Methods and Fig. 1A). We chose not to pursue globin depletion strategies with the rationale that this would increase the variability in the library preparation step. Instead, we implemented deeper sequencing of libraries to ensure sufficient gene detection in samples where globin mRNA is high.

### Using RNA-seq to reveal the impact of variants detected in disease genes obtained through prior genomic testing

Despite blood samples having been archived for 3-5 years, we obtained RNA libraries suitable for subsequent sequencing for all samples. The resulting RNA-seq libraries were sequenced to a median of 116 million reads with a median of 78% of the reads mapped uniquely to the hg19 genome. We detected a median of 12,071 genes (TPM > 1) and 176,284 junctions (>=5 reads supporting a junction) per sample across our cohort. A full list of quality control metrics for each sample can be found in Supplementary Data 1.

We focused on the 61 individuals for whom genomic variants in the initial studies and subsequent re-analysis had been considered as receiving a diagnosis^16–18^ (Group 1). These 61 cases were comprised of 52 cases with SNVs or indels, six cases with structural variants (SVs)/copy number variants (CNVs), and three cases with both candidate SNVs/indels and SV/CNVs. In 39 of the 61 cases, the disease-causing genes underlying the diagnosis were detectable in our blood RNA-seq data. RNA-seq revealed that in 20/39 cases the variants influenced transcription (altered expression, splicing, or allele-specific expression; Table 1, Fig. 1B) and the pathogenic classification of the DNA variants, as per ACMG guidelines, remains unchanged.

### RNA expression changes in previously identified disease genes

We examined the effect of SNVs or indels on gene expression for genes that we detected by whole blood RNA-seq. This variation included missense variants, splice site variants, frameshift or nonsense variants (Fig. 1C, Table 1, Supplementary Data 2). We found decreased gene expression levels in genes from 9 cases, including 5 cases with nonsense or frameshift variants associated with autosomal dominant (AD) Infantile Epileptic Encephalopathy with Severe Global Developmental delay (*PPP3CA*), autosomal recessive (AR) Alazami syndrome (*LARP7*), AD Mental Retardation Autosomal Dominant 19 (*CTNNB1*) and AR Diarrhea 10, protein-losing enteropathy type (*PLVAP*), AD ZTTK syndrome (*SON*) (Fig. 1C–F, Table 1).

RNA-seq also gave us an opportunity to assess the gene expression consequences of missense variants. While it is typically assumed that missense variants would not overtly influence gene expression levels, genome sequencing alone cannot determine this. We found that 19/21 of the diagnosed cases with a missense variant showed no obvious effect on gene expression. The genes from the two participants with a diagnostic missense variant showed decreased gene expression (z-score < -3) were *SLC25A19* (Case 11) and *BBS1* (Case 38) (Fig. 1C, Table 1). The *SLC25A19* result can be explained by the fact that in addition to a missense variant, the individual also possessed a heterozygous deletion that removes part of the last intron and the last exon of *SLC25A19* (Table 1). Case 38 did not have an initial explanation for the observed reduced *BBS1* expression, and the clinical data we used^16–18^ only reported the heterozygous pathogenic missense variant (NM_024649.5) c.1169 T > G (p.Met390Arg). However, additional consultation revealed that a subsequent genome reanalysis study at SickKids independently determined that a retrotransposon insertion occurred in trans to the heterozygous pathogenic missense variant, resulting in a premature termination codon^20^. Our RNA-seq analysis pipeline does not readily allow for the automated detection of such insertions and deletions. We did not initially flag these reads for further inspection due to the low expression of *BBS1* in blood. Manual inspection of the RNA-seq reads identified reads mapping to the exon and the retrotransposon sequence (Supplementary Fig. 2).

### Evidence for RNA splicing changes in previously identified disease genes

We observed splicing-related consequences in five individuals:

*Case 46*: aberrant splicing was present in an individual with a suspected mitochondrial disorder carrying a homozygous deletion occurring in the splice donor site of exon 5 of the *DNAJC19* gene (NM_145261.3) c.280+1_280+5delGTAAG which is associated with 3-Methylglutaconic Aciduria Type V. Interestingly, this variant results in two novel junctions, one causing the skipping of exon 5 and the other causing the skipping of both exon 4 and 5 (Fig. 1E). Both events are predicted to result in a frameshift. In addition, reduced expression of *DNAJC19* gene was also observed (Fig. 1D). While this variant was predicted to affect a canonical splice site, RNA-seq demonstrates a functional consequence of this deletion in vivo.

*Case 59*: aberrant splicing was confirmed in a case of Infantile Sialic Acid Storage disease where two rare variants in trans (confirmed by parental studies) were identified in the *SLC17A5* gene (NM_012434.4) c.819+1 G > A; c.291 G > A p.(Thr97 = ). Both variants were predicted to influence splicing by in silico predictions. RNA-seq analyses also confirm that aberrant splicing occurs and showed skipping of exon 2 (c.291 G > A p.Thr97 = ) and exon 6 (c.819+1 G > A p.?) (Fig. 1F). Both events are predicted to result in a frameshift. Moreover, these aberrant splicing events were associated with reduced levels of the *SLC17A5* transcripts suggesting the occurrence of nonsense mediated decay (NMD) (Table 1; Fig. 1D).

*Case 1008*: a rare heterozygous variant in the *SMARCB1* gene (NM_003073.3) c.364del (p.Glu122Asnfs*21) was previously identified in an individual clinically diagnosed with Coffin-Siris syndrome. This variant was predicted to cause a frameshift in this haploinsufficient gene. Our RNA-seq analysis did not reveal a significantly reduced expression of this gene (z-score: -1.53, fold change: 0.89, *p* value: 0.12). However, RNA splicing analysis showed a decreased usage of exon 4 via exon skipping (Supplementary Fig. 3A). Importantly c.364del (p.Glu122Asnfs*21) occurs adjacent to the splice acceptor site, two base pairs from the start of exon 4. Skipping of exon 4 results in an in-frame truncation of the SMARCB1 protein, the functional consequences of which remain unknown.

*Case 1016*: a homozygous frameshift deletion was identified in exon 2 of the *PANK2* gene (NM_153638.2) c.824_825del (p.Cys276Trpfs*15)) in an individual diagnosed with Neurodegeneration with Brain Iron Accumulation-1. We observed 88% (65/74 reads) of transcripts spliced to the short exon 2 of ENST00000336066, compared to only an average of 55% (152/272 reads) in ten randomly selected controls. Although short-read RNA-seq does not allow accurate measurement of isoform expression, estimation using RSEM suggested an increased usage of ENST00000336066 (65% vs. 52% cohort median). Long-read RNA-seq would likely help resolve the change in isoform usage^21^. While we only observed a small effect on the overall gene expression level (z-score: -1.97, fold change: 0.88, *p* value: 0.07; Supplementary Fig. 3B), the decreased usage of the transcript expressing the frameshift is likely due to NMD. The shift in usage to an isoform that skips the frameshift may be a compensatory mechanism for maintaining levels of *PANK2* expression. The impact at the protein level remains to be seen.

*Case 1049*: a heterozygous canonical splice site variant was identified in intron 6 of the *NSD1* gene (NM_022455.4) c.3922-1 G > C (p.?), which is associated with Sotos Syndrome. Our RNA-seq analysis detects two aberrant splicing events: The first involves skipping of exon 7 and increased usage of the resulting isoform (ENST00000375350), which is a frameshift splice isoform annotated as an ‘NMD transcript’ in the Ensembl database. However, our data shows that the *NSD1* variant increased the fraction of this isoform. Overall, the levels of *NSD1* expression were slightly higher in the proband relative to controls. Supporting this observation, a recent genome-wide screen for NMD escaping transcripts also identified ENST000000375350 as a NMD escaping frameshift variant^20^. The second splicing event involves the use of an alternative splice acceptor site 5 bp into exon 7 and is predicted to result in a frameshift (Supplementary Fig. 3C). The detection of the two aberrant transcripts suggests that they may be stable enough to be translated into a truncated protein with little residual or altered function.

### Evidence for allele-biased gene expression in previously assigned disease genes

We observed allele-biased expression in two individuals:

*Case 86*: A heterozygous missense variant in gene *SLC35A2* (NM_005660.1: c.991 G > A (p.Val331Ile)), associated with X-linked dominant Congenital disorder of glycosylation type I, showing allelic imbalance favoring the reference allele (45/69 reads, 76%). This could be due to skewed X-inactivation in different tissues, as has been previously reported^22^.

*Case 1103*: The two variants in trans in the *VPS53* gene (NM_018289.3) a c.1429 C > T (p.Arg477*) and a c.1716 T > G (p.Ser572Arg), are associated with Pontocerebellar hypoplasia, type 2E. We observed allelic bias towards the p.Arg477 reference residue 392/51 reads; 76%) and the p.Ser572Arg alternative allele (39/51 reads; 76%), respectively. This suggests that NMD of transcripts harboring the nonsense variant is occurring.

### Detecting gene expression outliers at disease-associated CNVs

**Fig. 2:**
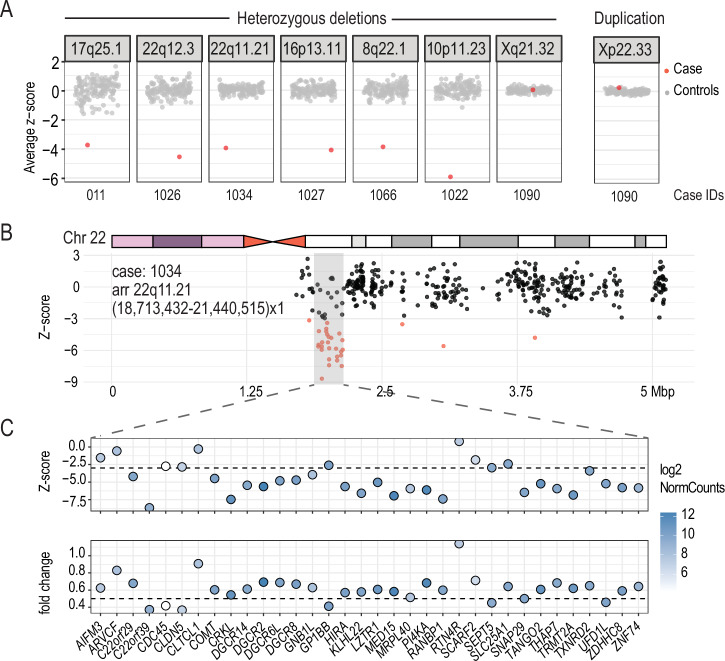
Modulation of gene expression by CNVs. **A** Z-score summary of genes within pathogenic CNVs. Y axis: average z-score of genes in pathogenic CNVs. Each dot represents one sample. Red: sample in which the pathogenic CNV is detected. Genomic locations of CNVs are shown as panel headers and case IDs are shown below. **B** Z-scores of all genes on chromosome 22 in case 1034. An **i**deogram of human chromosome 22 is shown. The heterozygous deleted region is highlighted in grey. Gene start is used as the proxy for gene location. Genes with an absolute z-score >= 3 are highlighted in red. **C** Expression changes of genes within the CNV. Dot plots show z-scores and fold-changes of detected protein-coding genes within the deleted region. Color represents the expression level (log2 normalized counts) of genes in this sample. Genes are ordered by genomic coordinates.

CNVs contribute significantly to rare diseases in human (e.g.^23–26^ and the effect of a CNV on gene expression can be detected in the general population, cancer and in rare diseases^27–34^. We examined the nine cases that carried diagnostic or partially diagnostic CNVs and asked if genes overlapping these CNVs show expression changes. In seven cases, these CNVs overlap one or more genes that are sufficiently detected in our cohort. Among these cases, six harbored heterozygous deletions that range from 4 kb to 1.3 Mb, overlapping 3 to 42 genes expressed in blood. In all six cases, we observed decreased expression of genes within the deleted regions as indicated by the decreased average z-score (compared to the rest of the cohort) of their expression (Fig. 2A). However, despite the consistent gene dosage change, the expression of genes within the same CNVs were not impacted uniformly (see Supplementary Data 3 for a list of outlier genes within CNVs for each sample). One individual with 22q11.2 deletion syndrome harbors the well-characterized heterozygous ~3MB deletion on chromosome 22 overlapping 137 genes. We focused on the expression of 34 protein-coding genes in this region and found that 24 of them showed decreased expression (z-score <= -3) with fold changes ranging from 0.37 to 0.69 (Fig. 2B, C, Supplementary Data 3).

Finally, the last CNV case (Case 1090) is an individual that carried complex X chromosome rearrangements (arr Xp22.33q21.32(60,701-91,873,757)x3, Xq21.32q28(91,877,172-155,174,078)x1) shows congenital anomalies, global developmental delay, severe intellectual disability, seizures, blindness, and deafness (Table 1). Additional clinical testing (G-banding and FISH analyses) performed on this individual indicates the presence of one normal X chromosome and one abnormal X chromosome. The abnormal X chromosome is an isodicentric X, which contains two copies of Xp22.33 to Xq21.3 with deletion of Xq21.3 to Xq28. No X chromosome material was located on another chromosome in the genome. Previous studies have shown that despite X chromosome copy number variations, most differentially expressed genes in individuals with Turner Syndrome are on autosomes (reviewed in ref. ^35^). To control for X chromosome copy number, we performed gene expression outlier analysis with OUTRIDER using female samples (*n* = 58) only. We first confirmed that this reanalysis did not change the observation of CNV-overlapping genes in the other cases (Supplementary Fig. 4A). Next, we re-examined gene expression changes in this individual. Consistent with previous reports, most genes with expression changes in case 1090 are not X-linked (7 X-linked genes /127 genes with absolute z-score > =3 or adjusted *p* value < 0.05, Supplementary Data 4). We found that X-linked genes within the duplicated region showed higher expression relative to the rest of the cohort, especially for p-arm genes distal to the centromere (Supplementary Fig. 4B, C). In contrast, the expression of genes within the deleted region is comparable to controls (Supplementary Fig. 4B, C), which could be explained by skewed X inactivation, which is known to occur with large structural changes on X chromosome^36^.

### Identifying splicing and expression outliers in a cohort with no previous molecular findings

There were 73 participants who despite traditional testing and reanalysis remained undiagnosed^16–18^. The clinical findings in this cohort included global developmental delay, epilepsy, and multiple congenital anomalies, connective tissue abnormalities, cardiomyopathy, suspected metabolic disorder, and ophthalmological or immune system abnormalities (see Supplementary Data 2). Using the information obtained from our RNA-seq analyses and discussions with clinicians, we identified plausible candidate genes related to the phenotype in five cases (Table 2):

**Table 2:** Candidate genes and additional variants detected in 7 case

Case	Primary indication for referral	RNA-seq Findings	DNA variant	Cohort
18	Global developmental delay and epileptic encephalopathy	*UGDH* decreased expression zScore: −4.15, FC: 0.62, *p* value: 7.50e−5	Not detected	Lionel et al.^17^
87	history of hypertrophic cardiomyopathy, short stature, developmental delay, and distinctive facies	*LZTR1* increased usage of an exon within intron 16 zScore: −1.55, FC: 0.85, *p* value: 1.09e-1	c.1943−351 G > C	Lionel et al.^17^
91	Global developmental delay, autism spectrum disorder, epilepsy, atrial septal defect	*PPP2CA* decreased expression zScore: −3.51, FC: 0.88, *p* value: 6.97e-4	Not detected	Lionel et al.^17^
1035	Constitutional overgrowth, autistic spectrum disorder, ADHD, pectus excavatum, advanced bone age	*NFIX* (Marshal or Sotos AD): increased usage of alternate transcript and NSD1 (Sotos): 9 bp shortening of exon 22	Not detected	Stavropolous et al.^16^
1058	ZTTK syndrome	*CEP120* decreased expression and increased usage of a splice isoform targeted for nonsense-mediated decay zScore: −4.74, FC: 0.76, *p* value: 4.15e−7	Not detected	Stavropolous et al.^16^
1085	Epilepsy, autism spectrum disorder, behavioral disorder	*PTK2B* creased expression and retention of intron 27 zScore: 5.97, FC: 1.68, *p* value: 1.56e−10	c.2523+1 G > A	Stavropolous et al.^16^
1101	Profound global developmental delay, spastic quadriplegia, Acute rhabdomyolysis	*B4GALT2* decreased expression zScore: −3.40, FC: 0.45, *p* value: 8.30e−4	Not detected	Stavropolous et al.^16^

**Fig. 3:**
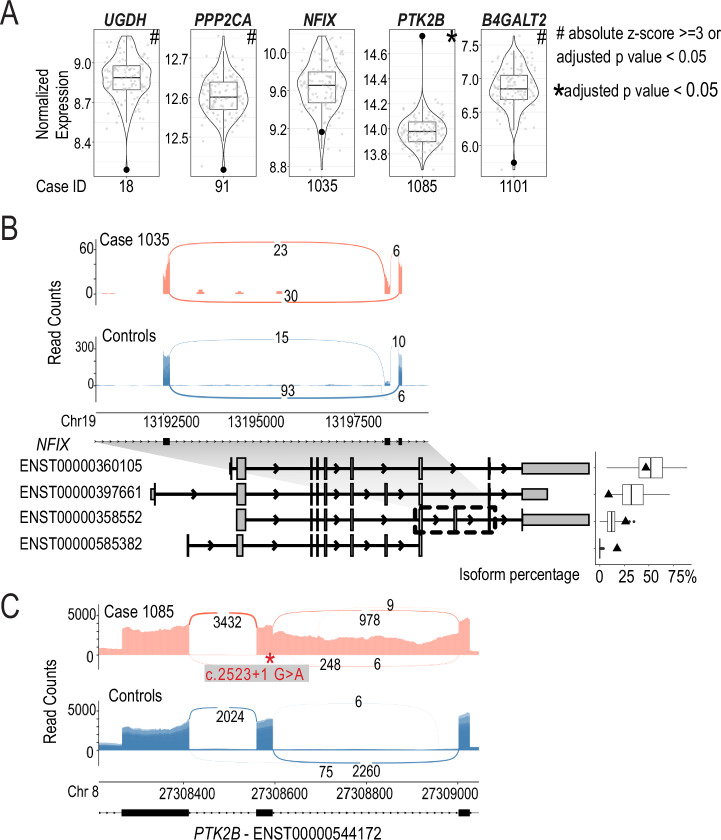
Aberrant splicing events and expression of candidate genes identified using RNA-seq. **A**. Violin and boxplot showing normalized gene expression of selected candidate genes across the cohort. Y axis: log2 normalized read counts of the target gene in all samples in the cohort. Each dot represents one sample. Highlighted dot represents the case of interest. Case IDs are shown below each plot. Number sign: the gene is considered an outlier in the analysis (absolute z-score >=3 or adjusted *p* value < 0.05); asterisk: the gene is detected as a significant outlier (adjusted *p* value < 0.05). **B**. Case 1035. Sashimi plot showing an increased exon usage event in *NFIX* in case (red track) and control samples (blue track, *n* = 10, randomly selected from the cohort). Bottom left: Structures of relevant transcripts. Exons shown in the sashimi plots are highlighted with a dashed box. Bottom right: Boxplot showing the percent isoform usage (“IsoPct” from RSEM output) of the corresponding transcript across the cohort. Each dot represents a sample. Isoform usage of the case is highlighted as a triangle. Only the transcripts detected in the case are shown. **C** Case 1085. Sashimi plot showing an intron retention event in *PTK2B*. The DNA variant is shown with an asterisk.

*Case 18*: Decreased expression of the *UGDH* gene, which is associated with AR Developmental and Epileptic Encephalopathy 84, was identified as an extreme outlier (z-score –4.15, fold change 0.62, *p* value 7.50e−05) in a male with global developmental delay and epileptic encephalopathy (Fig. 3A). The UGDH protein is involved in the biosynthesis of extracellular matrix components and loss-of-function variants result in epileptic encephalopathy with variable degrees of developmental delay^37^. No variant was detected in the GS data.

*Case 91*: Decreased expression of the *PPP2CA* gene (Protein Phosphatase 2, Catalytic Subunit, Alpha Isoform associated with AD Houge-Janssens syndrome 3) was identified as an outlier (z-score: −3.51, FC: 0.88, *p* value: 6.97e−4) in a female with global developmental delay, autism spectrum disorder, epilepsy, atrial septal defects (Fig. 3A). No variant was detected in the GS data.

*Case 1035*: Splicing outlier analysis identified increased usage of exon 9 of the *NFIX* gene (NM_002501) in the blood sample obtained from a male with constitutional overgrowth, autistic spectrum disorder, pectus excavatum, and advanced bone age (Fig. 3A,B). *NFIX* encodes a transcription factor and is associated with AD Marshall-Smith syndrome and Malan syndrome (Sotos syndrome 2), and is highly expressed in the brain cerebellum. No variant was detected in the GS data.

*Case 1085*: Splicing and expression analysis identified the *PTK2B* gene as a candidate gene due to an intron retention event in intron 27 in a male with features of autism, behavioral issues, obesity, epilepsy and growth hormone deficiency. This intron retention is predicted to result in a frameshift (NM_004103.4) (Fig. 3A, C). The *PTK2B* gene showed significantly increased expression (z-score 5.97, fold change 1.68, *p* value 1.56e−10). A re-examination of the GS data revealed a novel variant at +1 splice donor site of intron 27 (c.2523+1 G > A) that explains the intron retention. The function of the *PTK2B* gene is associated with signaling neuropeptide-activated receptors or neurotransmitters and may also provide a mechanism for a variety of short- and long-term calcium-dependent signaling events in the nervous system^38,39^. *PTK2B* has been identified as a risk factor for Alzheimer’s disease by multiple studies, and the mechanism of some susceptibility alleles has been attributed to splicing alterations^40–42^. One Alzheimer’s Disease-risk allele (rs28834970-C) present in this individual is associated with higher protein expression of PTK2B in monocytes^43^. In the GTEx whole blood expression dataset, rs28834970-C is also associated with moderately increased expression of *PTK2B*^44^. This raises the possibility that the individual’s phenotype is the result of two independent molecular events where the common disease-associated SNV (rs28834970-C) upregulates the expression of the aberrant frameshift containing transcript. Since it was not possible to phase these variants, the relevance of this proposed mechanism remains to be seen.

*Case 1101*: Decreased expression of the *B4GALT2* gene (UDP-GAL: beta-GlcNac beta-1,4-galactosyltransferase, polypeptide 2) gene was found in blood isolated from a male with profound global developmental delay, spastic quadriplegia and acute rhabdomyolysis (z-score: −3.40, FC: 0.45, *p* value: 8.30e−4) (Fig. 3A). Although the *B4GALT2* gene shares 55% amino acid identity with the *B4GALT1* gene, which is associated with Congenital disorder of glycosylation, type IId, the role of *B4GALT2* in disease is not known, and further analysis will be required to understand the possible role of the aberrant expression of the gene and the role it may play in relation to the phenotype of this individual. No variant was detected in the GS data. Overall, the application of RNA-seq to cases with no DNA findings resulted in candidate genes related to participant phenotypes in 5 out of the 73 cases.

**Fig. 4:**
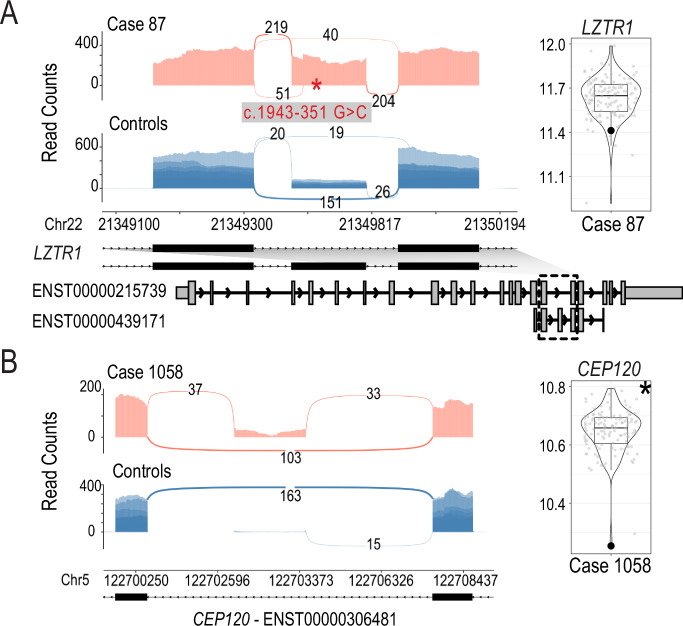
Aberrant splicing events and expression of candidate genes identified using RNA-seq. **A** Case 87. Sashimi plot showing the inclusion of a rarely used exon within intron 16 in *LZTR1* and violin plot showing the decreased expression of *LZTR1*. Models of relevant isoforms are shown below. **B** Case 1058. Sashimi plot showing an exon inclusion event in *CEP120* and a violin plot showing decreased expression of *CEP120*.

We identified two cases where RNA-seq provided an alternative diagnosis or additional variants of interest:

*Case 87*: In a male with history of hypertrophic cardiomyopathy, short stature, developmental delay, and distinctive facies in keeping with Noonan syndrome, we detected increased usage of a 117 bp exon within intron 16 of the *LZTR1* gene which is predicted to result in an in-frame insertion of 36 amino acids (NM_006767.4; Fig. 4A). Within this exon, we also detected an alternative splice acceptor site that is predicted to result in a premature stop site (Fig. A). We identified a homozygous rare deep intronic variant c.1943-351 G > C that is likely the cause of these two aberrant splicing events. All of the RNA-seq reads aligning to this exon originated from this variant. Although an *EPG5* variant had been detected by GS (Supplementary Data 2), this *LZTR1* finding was a stronger match to the individual’s clinical presentation and was considered diagnostic.

*Case 1058*: We detected a significant decrease in the expression of the *SON* gene (z-score −3.50, fold change 0.80, *p* value 6.00e−04) and no effect on splicing in blood obtained from a male who presented with global developmental delay, hypoplastic toes, syndactyly and macrocephaly (Fig. 4B). We also detected the *SON* (NM_138927.2) c.3476del (p.Pro1159Arg*9) variant, associated with AD ZTTK syndrome, which was independently identified during DNA re-analysis of the case^18^. This demonstrates the ability of an RNA-first approach to detect clinically relevant RNA events even if the DNA variant is not known at the time of analysis. We also identified another candidate gene *CEP120*, which is associated with Joubert syndrome^45^, with a novel exon between exons 18 and 19 (Fig. 4B) and decreased expression (z-score −4.54, fold change 0.76, *p* value 4.15e−7). No DNA variants were identified in the GS analysis. However, it has been demonstrated that a 57 bp deletion overlapping a pair of Tigger4 transposons within intron 17 causes expression of this novel exon^46^. We see evidence of this deletion in the RNA-seq data, but the zygosity is unclear (Supplementary Fig. 5). The SON protein regulates the splicing of centriolar protein genes, including *CEP131*^47^. It is possible that SON also regulates the splicing of *CEP120*, together with the *CEP120* intronic deletion, resulting in the observed reduction in gene expression and potentially contributing to the complexity of the individual’s phenotype.

## Discussion

In this study, we benchmarked the diagnostic utility of RNA-seq in a well-studied pediatric rare disease cohort (SickKids Genome Clinic) that was established for assessing the clinical utility of genome sequencing. This unique cohort allowed us to perform a follow-up assessment of blood RNA-seq focussing on two clinical testing scenarios. In the first scenario, RNA-seq was used to provide functional data for variants that had been identified by GS and were considered diagnostic for the participant’s phenotype. Using RNA expression as a functional readout of genetic changes is a rationale and practical way to help confirm diagnoses and suggest pathogenic mechanisms. Assigning a molecular mechanism to a genetic disease diagnosis is a requisite for bespoke genetic therapy development^48^. In the second scenario, we aimed to identify candidate genes using a transcriptome-first analysis. Our work showcases a variety of cases where RNA-seq effectively facilitates the diagnosis and will contribute to the establishment of a clinical guideline.

In the first scenario, when comparing the RNA-seq data to in silico prediction algorithms (SpliceAI for splicing impact, Supplementary Data 2), the results are concordant for the majority (29/34 cases) of the known pathogenic or likely pathogenic SNVs and indels analyzed. The five cases that appeared to be discordant benefited from a manual inspection of the RNA-seq data. First, we predicted that the homozygous c.2600_2601delTA p.(Leu867*) [NM_017733.3] variant in the *PIGG* (Case 64) gene would result in decreased expression. We only observed moderately decreased transcript levels (z-score −2.34, fold change 0.88, *p* value 0.028). While this decreased expression is suggestive of NMD, the gene expression reduction did not pass our cutoff filters. This illustrates the impact that gene expression cut-offs and filtering algorithms will have on potentially biologically complex, yet still meaningful observations. A combined approach of lenient cutoffs for well-studied genes and more stringent cutoffs for novel disease genes could help alleviate this issue.

Next, one missense variant, (Case 1029*: PIK3R2* c.1117 G > A (p.Gly373Arg) [NM_005027.4]), 7 bp from the splice acceptor sites was computationally predicted to cause an acceptor loss 2 bp from the variant while RNA-seq showed no impact. Upon closer examination, the predicted loss does not occur at a splice site used in the population and is thus likely a false positive prediction. Similarly, SpliceAI failed to predict the splicing impact of the *PANK2* (Case 1016) variant. However, another tool, Pangolin^49^, predicted the alternative splice site usage (a donor loss (score = 0.28) and splice donor gain (score = 0.19)) observed with the RNA-seq data. In addition, the frameshift variant in the *SMARCB1* (Case 1008) gene was not predicted to be splice-altering but we showed a splicing impact. Finally, in Case 46, SpliceAI only reported the most proximal splicing change (the skipping of exon 5) and did not predict the more complicated event (skipping of exon 4). While our analyses show that in silico approaches can fail to predict the specific transcriptional outcome, getting a consensus result from multiple tools will improve the performance of computational prediction. RNA-seq data provides individual-specific, empirical information on specific splicing mechanisms (exon skip, intron retention), which can be useful even when canonical splice site variants are encountered. This observation echoes the conclusions from other published work^50^ and highlights the need for obtaining more pediatric RNA-seq data to improve in silico RNA splicing prediction algorithms.

Our study also highlights the value of using RNA-seq in cases with large CNVs identified by CMA. Consistent with existing studies, we showed that gene expression changes cannot be accurately predicted from gene copy number (identified by array or GS) alone^27,28,30,32,33^. The examples that we present show that RNA-seq provides information on dosage compensation at the transcriptional level for individual genes within CNVs and complex chromosomal rearrangements. For example, decreased dosage of a combination of genes in individuals with 22q11 deletion syndrome is attributed to the complex and variable 22q11 deletion syndrome phenotypes^51,52^. By measuring the expression change of every gene in an unbiased manner, RNA-seq could provide further insights into the pathogenicity of CNVs and pinpoint sets of genes contributing to inter-individual differences in phenotype. These data will be relevant for the more complex phenotypes and add a nuanced interpretation to genome sequencing findings. However, additional information on gene expression and epigenomic features in disease-relevant and developmental stage-specific samples will be needed to understand the pathogenicity of sets of genes affected by CNVs^27,33,53^.

Using an RNA-centric approach on the second cohort of individuals who did not receive a genetic diagnosis by GS or CMA, we identified aberrant splicing or expression events related to the primary clinical indication in ~10% (7/73) of the cases, including a diagnostic variant in *LZTR1* (case 87) before a DNA variant had been found, underscoring the ability of RNA-seq to improve genome diagnosis. However, it remained challenging to identify a causal DNA variant leading to these clinically relevant RNA phenotypes, especially for gene expression outliers. The yield seen in this RNA-centric approach may be the result of DNA variants that are poorly annotated, for example, regulatory, intronic or intragenic areas of the genome, or structural changes not identified by short-read sequencing.

Both the genomic and transcriptomic analyses focused on genes with known disease or HPO associations, which will limit our ability to identify molecular changes in novel genes. During this study our clinical lab utilized the hg19 as the reference genome, and we acknowledge the importance of reanalysis on newer genome assemblies. For our study anticipate a small impact of a genome assembly update, as none of the genes we identified in this manuscript were among those shown to be impacted by differences in genome build (hg19 vs hg38^54^). Additionally, our splicing pipeline examines de novo identified junctions independent of gene annotations. However, it is still possible that an updated genome build, and updated disease gene annotations could reveal additional diagnoses. As a first step to rule out changes, we processed all RNA-seq samples using hg38 (Ensembl gene annotation v104). We re-examined all outlier gene expression events using OUTRIDER and reproduced Fig. 1C. Despite slight differences in z scores and *p* values, the overall findings remained the same (Supplementary Fig. 6).

Our study highlights some of the challenges facing the routine use RNA-seq in a clinical genetic setting identified in previous studies^4–15^. Firstly, using blood as a proxy tissue for non-blood related clinical indications may fail to detect gene expression levels and cell-type specific splicing. Even in the cases where robust gene expression was detected in blood, different epigenetic processes in the disease-relevant cell type could change the outlier gene expression and splicing results, which in turn could change the clinical interpretation. More sensitive targeted technologies such as targeted PCR-based approaches (e.g., ref. ^7^), long-read RNA-seq approaches^21,55^, as well as trans-differentiation of accessible cell types from blood or skin into more disease-relevant tissue types (e.g., ref. ^56^) will help to overcome some of these challenges. While we have used our own cohort as a pediatric control population to minimize technical and biological differences, subtle variations may have escaped the identification of outliers under a fixed cutoff. Since the number of controls has a strong impact on gene expression outlier analysis^11^, it is critical to collect age-, sex-, and tissue-matched control cohort that is processed using a uniform experimental and computational pipeline. Building and sharing databases of high-quality pediatric RNA expression data is a realistic solution to achieve this.

In conclusion, our data show the utility of peripheral blood RNA-seq in assessing the genetic underpinnings of a diverse range of disorders. By utilizing a clinical-grade, automated version of a standard RNA-seq library preparation methodology on whole blood samples, we show that RNA-seq is sufficiently sensitive and reproducible to provide functional evidence for variants identified by genome sequencing. In this context, our results support using RNA-seq diagnostics in parallel with genomic sequencing. Such benefits could include increasing confidence in, and reducing the time spent reporting GS findings. An immediate application for RNA-seq is to provide functional evidence for variants currently classified under ACMG guidelines as variants of uncertain significance (VUS), which may lead to re-classification to likely benign/benign or likely pathogenic/pathogenic. Furthermore, providing critical clarification of variant impact, a critical step for assessing and designing individualized genetic medicines^48^. However, from a health economics perspective, it is unclear if a dual DNA/RNA testing regime is financially justified and how it compares to reserving RNA-seq for individuals for whom GS did not result in a conclusive finding. Nevertheless, expediting the production of a comprehensive and diverse reference set of pediatric blood gene expression profiles will enhance RNA-based genetic diagnoses and enable new algorithms for precision child health.

## Methods

### Cohort description

The cohort in this study was derived from the SickKids Genome Clinic, which consisted of two studies that collected samples over a 2-year period (April 2013 to June 2015). The participants presented with a spectrum of complex clinical indication,s including epilepsy, global developmental delay, and multiple congenital anomalies, which represent the most common indications for genetic testing. The SickKids Genomic Clinic participants first underwent genetic testing that met the standard of care provided at the time and were subsequently examined using genome sequencing. The first study cohort (which compared the diagnostic rate of genome sequencing to traditional chromosomal microarray) involved 100 children with suspected genetic disorders^16^. The second cohort (which compared genome sequencing to targeted gene panels) involved 103 children^17^. Variants were classified using ACMG guidelines^19^. In silico predictions for variants impact were generated using Ensembl VEP and SpliceAI, which was accessed through SpliceAI lookup (https://spliceailookup.broadinstitute.org/). SpliceAI prediction results are included in Supplementary Data 2.

### Research ethics

This study was approved by the Research Ethics Board at The Hospital for Sick Children, and informed consent was obtained for all participants (REB number 1000037726). Case IDs are de-identified and are not known to anyone outside the research group. We have complied with all relevant ethical regulations, including the Declaration of Helsinki.

Individual’s phenotypic data used in this study, to prioritize splicing and differential expression events, were previously captured in PhenoTips (http://www.phenotips.com)^57^. Phenotypic information is represented using the Human Phenotype Ontology (HPO)^58^. Participant data also included data regarding molecular genetic testing, including single gene or gene panels, extracted from the electronic medical records. Chemistry tests (blood and urine), enzymatic studies, muscle biopsies, and medical imaging, when available, were also included as previously described by both Stavropoulos et al.^16^ and Lionel et al.^16,17^.

### RNA isolation, library preparation, and sequencing

RNA from the 134 probands was extracted from blood collected in PAXGene tubes using an automated QIAsymphony PAXGene blood RNA kit (Qiagen). RNA was quantified using the Qubit Fluorometer (Thermo Fisher Scientific) High Sensitivity Assay, and sample purity was checked using the Nanodrop (Thermo Fisher Scientific) OD 260/280 ratio. Following the manufacturer’s recommended protocol, 250 ng of total RNA spiked with the Spike-in RNA variants of the isoform (SIRV) Set 3 (Lexogen), such that spike-ins were ~1% of the mRNA, were used as input material for library preparation. SIRV set3 includes 92 ERCCs and 69 SIRV isoforms corresponding to seven SIRV genes, allowing the assessment of an RNA-seq platform’s ability to detect a range of RNA concentrations as well as isoform complexity. Libraries were prepared using an automated NEBNext Ultra II Directional Library Prep Kit for Illumina with polyA isolation at Genome Diagnostics (Department of Laboratory Medicine, The Hospital for Sick Children). Libraries were assessed using the Bioanalyzer DNA High Sensitivity chips (Agilent Technologies, Santa Clara, CA) and quantified by quantitative polymerase chain reaction using the Kapa Library Quantification Illumina/ABI Prism Kit protocol (KAPA Biosystems, Roche, Basel, Switzerland). Libraries were pooled in equimolar quantities and paired-end sequenced on an Illumina NovaSeq6000 system, following Illumina’s recommended protocol, to generate paired-end reads of 150 bases in length at The Center for Applied Genomics, The Hospital for Sick Children, Toronto, Canada. A median of 116 million paired-end reads per library was obtained.

### RNA-seq data analysis and annotation

A customized RNA-seq processing pipeline was used for read alignment, quality control (QC), identification of expression and splicing aberrations, and variant calling. Raw sequencing reads were aligned using STAR^59^ (v2.6.1c) to the combined genome of hg19 (1000 genomes reference genome, hs37d5) and the spike-in sequences (SIRVome, SIRV set3). Gene annotation was obtained from Ensembl GRCh37 assembly archive (v87) and was combined with SIRVome transcript annotation obtained from the manufacturer (https://www.lexogen.com/sirvs/download/). Additional gene-disease association annotations were obtained from various sources: OMIM gene accession IDs and disease descriptions (Ensembl v87); HPO gene-disease association (https://hpo.jax.org/data/annotations); numbers of ClinVar variants for each gene (https://ftp.ncbi.nlm.nih.gov/pub/clinvar/tab_delimited/gene_specific_summary.txt); and Orphanet (http://www.orphadata.org/) in November 2019. Fastqc^60^ (v0.11.5), RNA-seQC^61^ (v2.3.5), Picard (https://broadinstitute.github.io/picard/) (v2.18.0), Markduplicates and CollectRnaSeqMetrics, supplemented with customized script,s were used to collect various QC metrics. Gene and transcript expression level quantification was performed using RSEM^62^ (v1.2.22).

### Gene expression outlier analysis

Gene expression outliers were identified by comparing each sample to the rest of the samples in the cohort using R package OUTRIDER^63^ (v 1.8.0). First, to minimize the effect of variable hemoglobin content, we removed nine hemoglobin genes (H*BB, HBD, HBG1, HBG2, HBZ, HBM, HBA2, HBA1, HBQ1*). Next, gene read counts estimated by RSEM were filtered for lowly expressed genes (only genes with a 95th percentile RPKM >1 were used). OUTRIDER results for samples 230373, 230577, and 239099 were not analyzed as they were identified as outliers after PCA analysis using OUTRIDER normalized counts (Supplementary Fig. 1). Subsequent examination showed these samples have suboptimal library quality. For each sample, genes with either an adjusted *p* value <0.05 or an absolute z-score ≥ 3 were reported as expression outliers and further examined manually.

### Splicing outlier analysis

To identify aberrant splicing events, we adopted the approach described by Fresard et al.^11^. Briefly, we used junction quantification by STAR (*SJ.out file). Only junctions with more than 5 uniquely mapped reads were considered in the analysis. Next, we calculated a junction coverage score, defined as the number of reads mapping to a junction of interest divided by the total number of reads mapping to other junctions that share a splicing donor or acceptor site with the junction of interest. We then calculated the z-score of the junction coverage score for each junction within the study cohort. Junctions with an absolute z-score ≥ 2 were further examined.

In addition, we also applied the aberrant splicing module from the Detection of RNA Outlier Pipeline (DROP)^64^ with default settings as a complementary approach to identify aberrant splicing events. Specifically, the DROP pipeline uses the R package FRASER^65^, which assesses the statistical significance of aberrant splicing events in addition to reporting z-scores, providing additional criteria for prioritization. Splicing events were visualized using ggsashimi^66^. Gene models were visualized using the R package ggtranscript^67^ based on Ensembl hg19 (v87) gene annotation. Introns were shortened using the function shorten_gaps().

### Allele-specific expression

Allele-specific expression (ASE) events were detected using a method adapted from the “MAE” module of the DROP pipeline. Briefly, for each sample, heterozygous SNPs identified from GS were first filtered for quality as previously described^16,17^ and further filtered to retain variants that are only identified in one sample in our internal batch. “ASEReadCount” from GATK^68^ (v4.0.1.2) was used to count the number of RNA-seq reads mapping to reference or alternative alleles of filtered SNPs. DESeq2^69^ (v1.44) was then used to test for significant bias towards reference or alternative alleles. ASE events with an alternative allele read ratio >0.8 and adjusted *p* values < 0.05 were further examined.

### Interpretation workflow

Splicing junctions were prioritized first using technical filters (e.g., z-scores, number of junction reads, and junction coverage ratio) followed by the biological significance of the genes containing the candidate junctions. Specifically, genes with HPO terms corresponding to each participant’s disease presentation were prioritized, followed by genes with any disease associations, and genes with no known disease associations. All splicing junctions that were considered clinically significant were visually inspected using the Integrated Genomics Viewer (IGV)^70^. Expression outliers and ASE genes were prioritized using z-scores and the adjusted *p*-value for each sample. Similarly, genes with relevant HPO terms or disease associations were prioritized.

## Supplementary information

Supplementary Materials

Supplementary Data 1

Supplementary Data 2

Supplementary Data 3

Supplementary Data 4

## Data Availability

The data supporting the current study was consented for research but not for sharing in public databases. Based on the results of our study this dataset will serve as pediatric blood expression control samples for rare disease research. Please contact the corresponding authors if you wish access to this data.

## Code availability

The RNA-seq processing pipeline and scripts for analyzing data and generating plots have been deposited on Zenodo (10.5281/zenodo.14889940).
