# Supplementary material for: Assessing the diagnostic impact of blood transcriptome profiling in a pediatric cohort previously assessed by genome sequencing
Source: NPJ Genom Med. 2025 Jul 1;10:51. doi: 10.1038/s41525-025-00505-4 (PMC12215727; doi:10.1038/s41525-025-00505-4)
Supplement: Supplementary file 1 — Supplementary Materials [file 41525_2025_505_MOESM1_ESM.pdf]

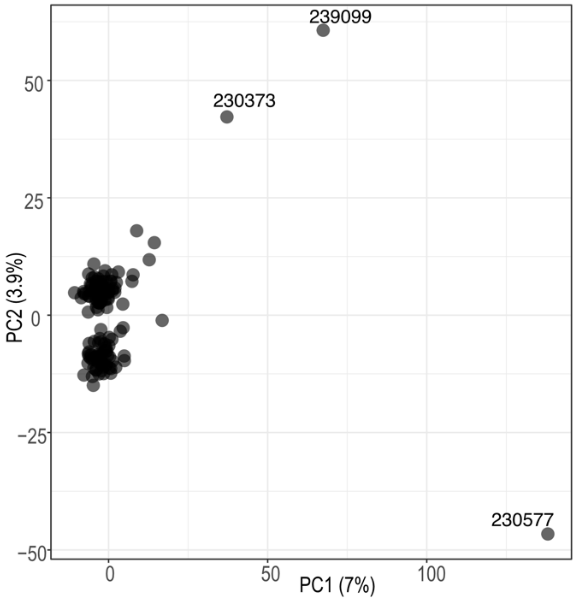

Supplementary Figure 1. PCA plot showing outlier samples after OUTRIDER normalization. Each dot represents one sample. The IDs of the three outlier samples are shown. Log2 transformed "normcounts" from OUTRIDER output was used for the PCA analysis.

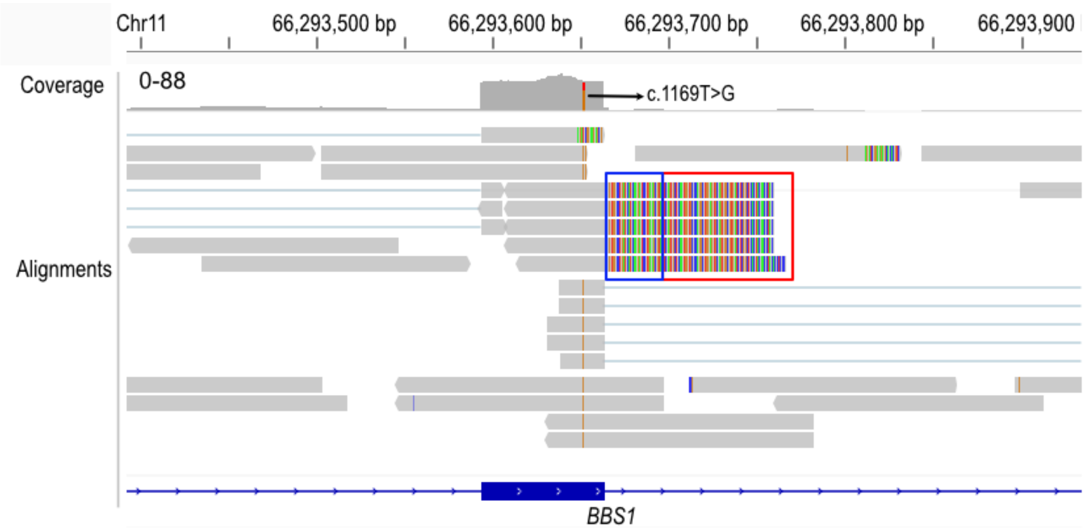

Supplementary Figure 2. IGV screenshot showing reads with retrotransposon insertion mapped to *BBS1*. From top to bottom: 1) genome scale; 2) read coverage track with the scale bar shown on the side; the pathogenic variant is indicated by an arrow; 3) subset of aligned reads. Red box shows the retrotransposon insertion sequences and blue box indicates sequences that should have been mapped to exon 13. 4) gene annotation track showing the exon 12 of *BBS1*.

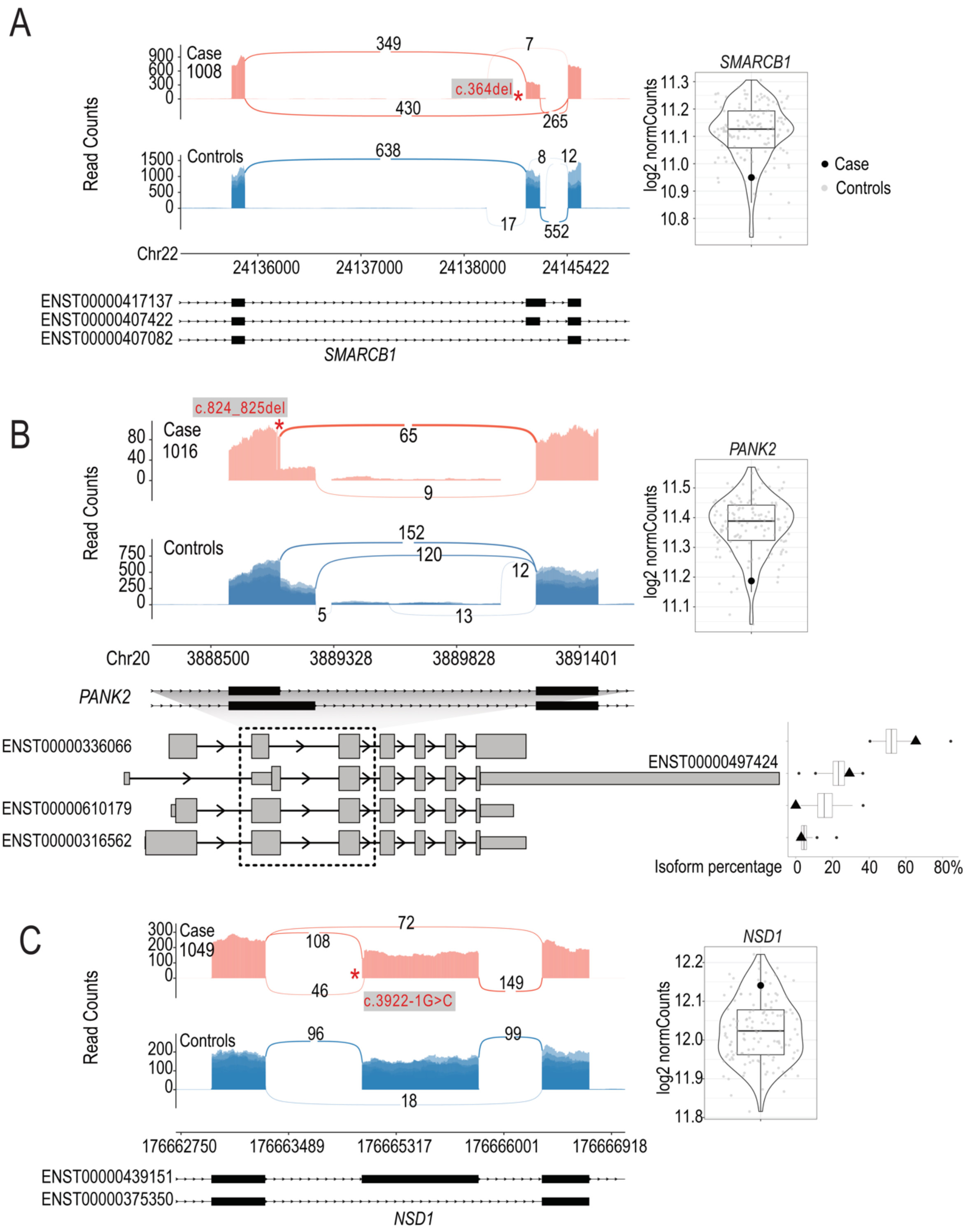

Supplementary Figure 3. Transcriptional impact of candidate variants identified with WGS/WES. A. Sashimi plot showing an exon skipping event in gene *SMARCB1.* Red: proband; blue: control samples (n = 10, randomly selected). Y axis: read counts. Violin plot showing non-significant decreased expression of *SMARCB1*. Y axis: log2 normalized read

counts. Black dot: cases in which the corresponding gene was a candidate; Grey dot: rest of the cohort as control samples. The DNA variant is shown with an asterisk **B**. **Sashimi plot showing increased usage of a shorter exon in gene *PANK2* and the pathogenic variant (two base pair deletion).** Violin plot showing non-significant decreased expression of *PANK2*. Bottom left: Structures of relevant transcripts. Exons shown in the sashimi plots are highlighted with a dashed box. Bottom right: Boxplot showing the percent isoform usage ("IsoPct" from RSEM output) of the corresponding transcript across the cohort. Each dot represents a sample. Isoform usage of the case is highlighted as a triangle. **C.** **Sashimi plot showing increased usage of an isoform that skips impacted exon and 5bp shift of the impacted splice acceptor site in gene *NSD1*.** Violin plot showing no notable impact on *NSD1* expression.

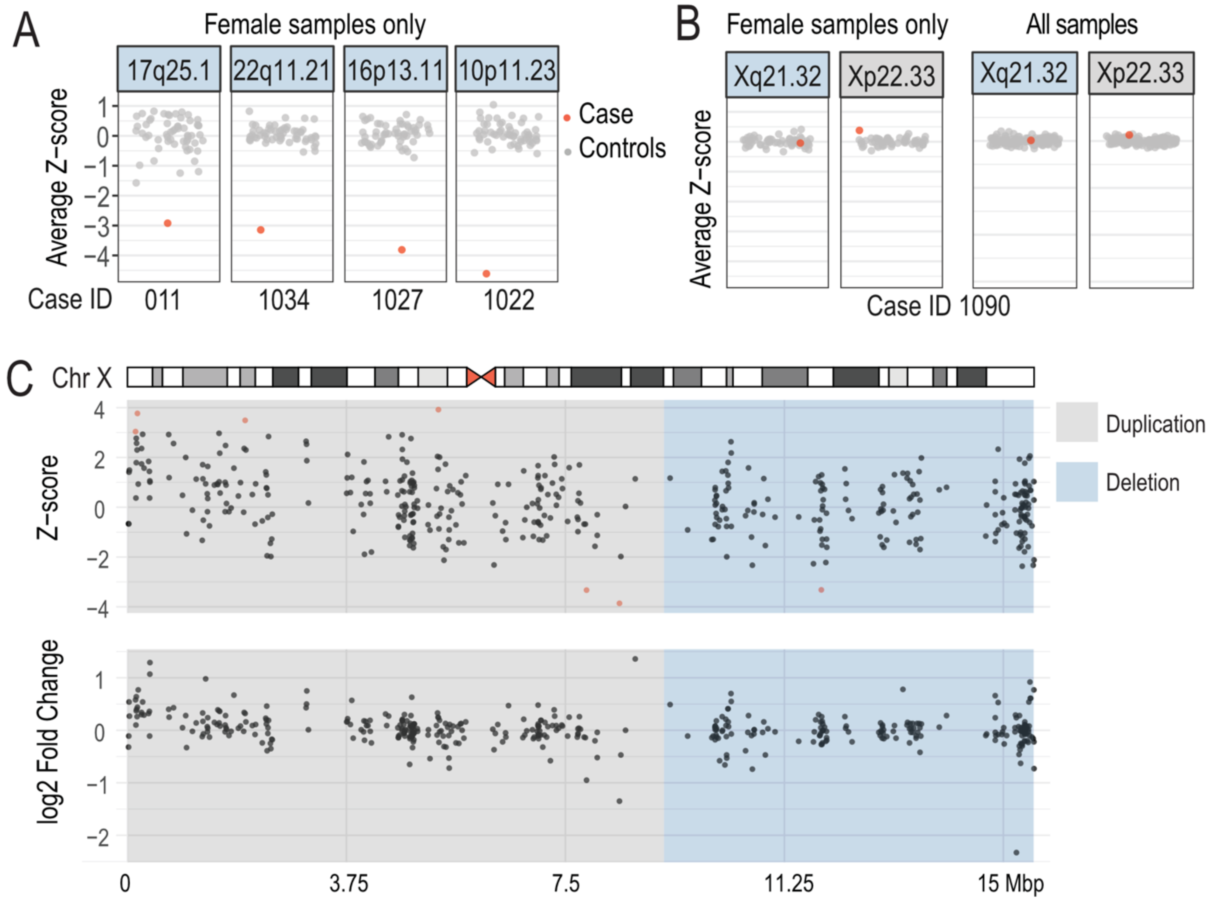

Supplementary Figure 4. Modulation of gene expression by CNVs analyzed using female samples only. Z-score summary of genes within **A.** pathogenic autosomal CNVs and **B.** chromosome X CNVs. Analysis performed with female samples only or all samples. Y axis: average z-score of genes in pathogenic CNVs. Each dot represents one sample. Red: sample in which the pathogenic CNV is detected. Panel header: Sample ID and CNV coordinate. C**. Z-scores and fold-changes of all genes on chromosome X in case 1090.** An Ideogram of human chromosome X is shown. The heterozygous duplicated region is highlighted in grey and deleted region in blue. Gene start is used as the proxy for gene location. Genes with an absolute z-score >= 3 are highlighted in red.

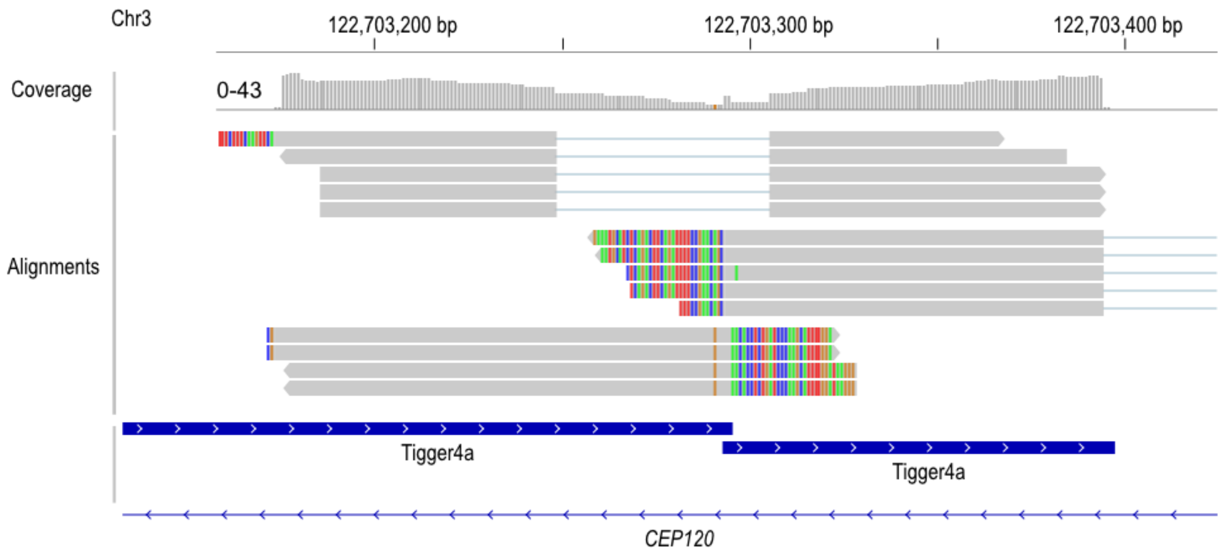

Supplementary Figure 5. IGV screenshot showing read alignment and coverage across the novel exon in *CEP120*. From top to bottom: 1) genome scale; 2) coverage track with the scale bar shown on the side; 3) selected aligned reads showing the 57 bp deletion (first group) and misalignment due to repetitive elements (second and third groups). Colored bases represent soft-clipped bases. 4) transposable elements and gene model track.

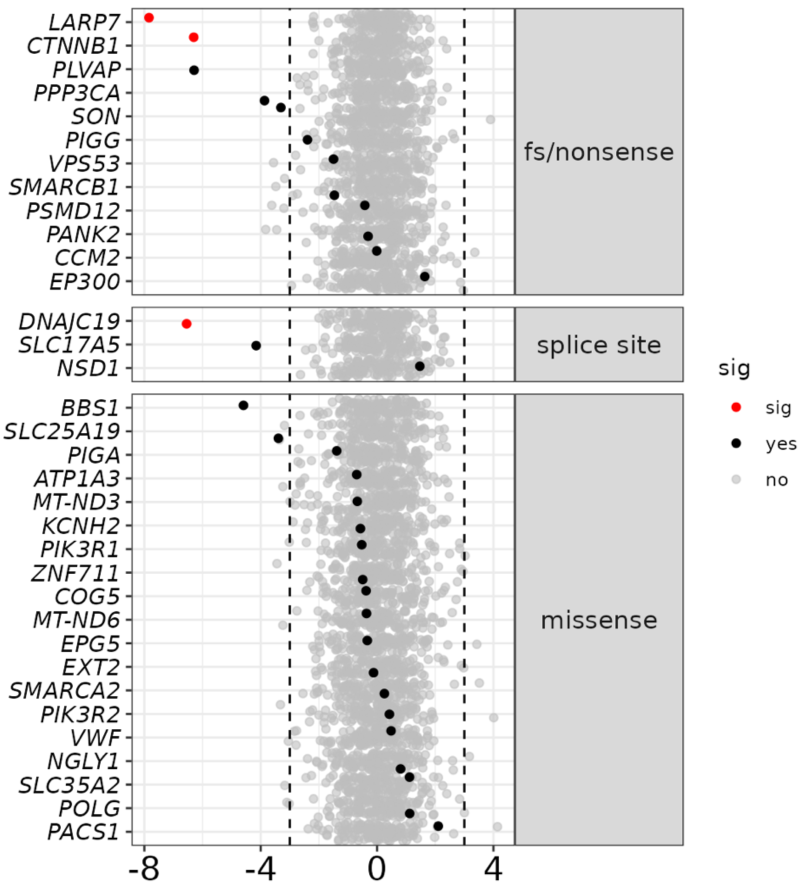

Supplementary Figure 6. Regeneration of main text Figure 1C (which used hg19) using the hg38 genome assembly. Detection of genes with diagnostic variants as expression outliers. Each row represents one gene. Each dot represents the z-scores of the corresponding gene in all samples. Black: cases in which the corresponding gene had a diagnostic variant; Grey: rest of the cohort as control samples. Asterisks indicate that the candidate gene is also detected as significant (adjusted p value < 0.05). Dashed lines indicated z-score of –3 or 3. Genes are separated into different panels based on the type of mutations they harbor.

Supplementary Data 1. Quality control metrics for RNA quality, RNA-seq data, and Spike-in controls.

Supplementary Data 2. Table of phenotypes for all individuals in this study.

Supplementary Data 3. List of outlier genes within CNVs for each sample using all samples as controls.

Supplementary Data 4. List of outlier genes within CNVs for each sample using only female samples as controls.
